# The magnitude of intimate partner violence during pregnancy in Eldoret, Kenya: exigency for policy action

**DOI:** 10.1093/heapol/czaa103

**Published:** 2020-11-09

**Authors:** Loice Luhumyo, Emily Mwaliko, Philliph Tonui, Amos Getanda, Katrina Hann

**Affiliations:** 1 Consultant Obstetrician Gynaecologist, Uasin Gishu County, Kenya; 2 Department of Reproductive Health, Moi University, Box 4606, Eldoret, Kenya; 3 Department ofMidwifery and Gender, Moi University School of Nursing, Box 4606, Eldoret, Kenya; 4 Sustainable Health Systems, Freetown, Sierra Leone

**Keywords:** Intimate partner violence in pregnancy, prevalence, determinants, perinatal outcomes, policy action, screening in pregnancy

## Abstract

Intimate partner violence (IPV) is sexual, psychological and physical coercive acts used against persons by intimate partners. When IPV occurs during pregnancy (IPVp), it can result in adverse maternal and pregnancy outcomes. No policy nor practice direction exists to address the rates and risk factors of IPVp in Kenya. Determining the prevalence, types and determinants of IPVp in Western Kenya would aid in the identification of pregnant women affected by and/or at risk of IPVp, as well as informing the development of policy, practices and programmes to support preventive interventions. In this cross-sectional study of 369 women who had given birth at Moi Teaching and Referral Hospital, participants were recruited using systematic sampling and data collected via structured questionnaires adopted from the WHO Violence Against Women Instrument. Associations were made in relation to physical or sexual violence and psychological violence. Logistic regression was used to assess the association between determinants and occurrence of IPVp. The overall prevalence of IPVp was 34.1%. Prevalence of physical or sexual violence was 22.8%. Psychological violence emerged as the most common (27.4%) form of IPVp. A lower than tertiary level of education and previous experience of IPV were individually associated with physical/sexual IPVp, whereas psychological IPVp was associated with previous experience of IPV and was prevented by the intimate partner having formal employment. Preterm birth rates were found to be higher than the country’s rates. The prevalence rates of IPVp are high in Western Kenya. Strategies that address the promotion of respectful, nonviolent relationships and that interrupt the development of risk factors are required. Policies (clinical guidelines) targeting prevention of IPVp and screening and the identification of at-risk women and survivors of IPVp are needed urgently. Primary prevention through interrupting the occurrence of predisposing factors is key in addressing IPVp.

**KEY MESSAGES**

Intimate partner violence in pregnancy (IPVp) remains highly prevalent in Western Kenya, with psychological IPVp as the most common type.
A previous history of intimate partner violence is strongly associated with physical/sexual and psychological intimate partner violence in pregnancy.
Prematurity is the most frequent perinatal outcome for the survivors of IPVp.
Policies, practices and programmes to promote respectful and nonviolent relationships are needed to interrupt the development of perpetration and address predisposing factors of IPVp.

## Introduction

Violence against women (VAW) is a global public health problem and a violation of fundamental human rights. The prevalence of VAW varies not only from country to country but also within countries. It takes many forms, including honour killings, early marriages, trafficking, female genital mutilation and intimate partner violence (IPV) (WHO, 2013).

Globally, most of the documented VAW is IPV (WHO, 2017). IPV against women is defined as the range of sexually, psychologically and physically coercive acts of violence and threats of such acts used against adult and adolescent women by current or former male intimate partners (Saltzman *et al.*, 2002). An intimate partner is one who has/had a close relationship with a person characterized by emotional connectedness, regular contact, ongoing physical contact, sexual behaviour, identity as a couple and familiarity with and knowledge about each other’s lives (Breiding, 2015).

In Kenyan law, IPV is captured in the Protection Against Domestic Violence Act (2015), where it is part of domestic violence by virtue of occurrence of sexual violence within marriage, emotional or psychological abuse, verbal abuse, stalking, physical abuse, harassment or any other conduct against an intimate partner where such conduct harms or may cause harm to the safety, health or well-being of the person. The Kenyan constitution of 2010 provides for the security and protection of a person against all forms of violence, with the article stating that every person has a right to freedom and security, which includes the right not to be subjected to any form of torture (Kenya, the Constitution 2010).

IPV is a common occurrence in the region. In East Africa, the prevalence of IPV ranges from 13.5% in Uganda (Devries *et al.*, 2010) to 39% in Tanzania (Msuya *et al.*, 2014). In Kenya, 38.0% of married women have experienced physical IPV, whereas ∼14% have experienced sexual IPV (KNBS & ICF, 2015).

In a survey carried out on 6002 households, pregnant women’s risk of abusive violence was found to be 60.6% greater than that of non-pregnant women (Newberger *et al.*, 1992). In 2013, a study conducted in Kisumu District Hospital in Kenya found an IPV prevalence rate of 37% among pregnant women attending the antenatal clinic (Makayoto *et al.* 2013). Another study of antenatal mothers in West Pokot County in Kenya found a higher prevalence of intimate partner violence during pregnancy (IPVp) of 66.9% (Owaka *et al.*, 2017). Compared with physical and sexual violence, psychological violence is found to occur most commonly in several studies in the region (Hoque *et al.*, 2009; Makayoto *et al.*, 2013; Groves *et al.*, 2015; Owaka *et al.*, 2017).

Despite growing evidence in this field, current estimates of IPVp are likely to underestimate the scale of the problem. This happens because of shame and fear of retaliation by the perpetrator and/or lack of standardized methods for its diagnosis or measurement (Enrique, 2004).

**Figure 1:**
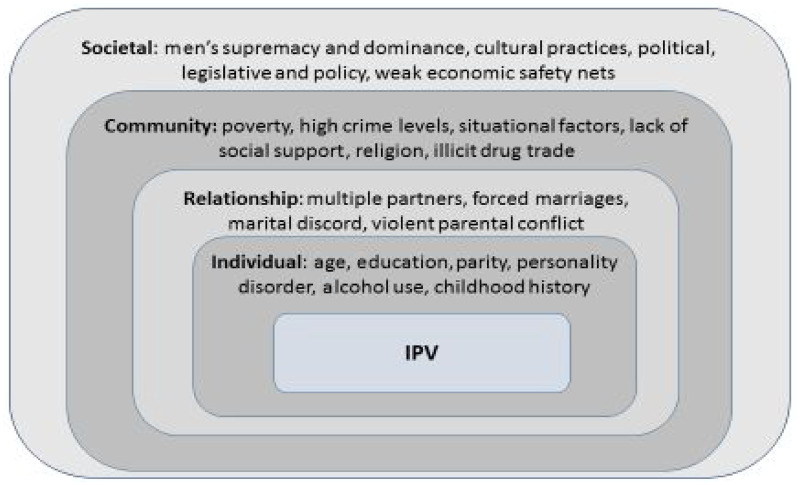
Determinants of IPVp in a socio-ecological model. Adapted from (WHO, 2018) 'The ecological framework of IPV: examples of risk factors at each level', www.who.int, http://www.who.int/ violenceprevention/approach/ecology/en/

IPV is a multifaceted phenomenon which is as a result of factors operating at different levels. The ecological framework of IPV classifies these factors as Individual, Relationship, Community and Societal (see Figure 1). These factors include but are not limited to a history of childhood abuse, alcohol and drug use, socio-economic status, exposure to violence while young, weak legal sanctions, cultural norms and male dominance (WHO, 2012). This framework helps to look closely at determinants of IPVp, therefore enabling interventions at multiple levels.

Whenever it occurs, IPVp involves a range of immediate and long-term complications to the mother and foetus. It has been associated with poor obstetric outcomes such as inadequate antenatal care, vaginal bleeding, hypertension, pre-eclampsia, abortions, depression and unintended pregnancies (Han and Stewart, 2014).The psychological effects are more subtle than the physical effects. They range from fear, anxiety, fatigue, post-traumatic stress disorder and sleeping and eating disturbances, which are more common long-term reactions to IPVp (Heise *et al.*, 1994).

IPVp has also been documented to cause adverse perinatal outcomes. A cross-sectional survey of women attending family practice clinics in Columbia, South Carolina, that was investigating the association of partner physical or emotional abuse during pregnancy and pregnancy outcomes, concluded that abuse during pregnancy was directly or indirectly associated with an increased risk of perinatal death, preterm delivery and low birth weight (LBW) (Coker *et al.*, 2004). More so, a systematic review conducted by Han and Stewart in 2014 concluded that IPVp was associated with prematurity, LBW, stillbirth and neonatal complications.

Local studies (Makayoto *et al.*, 2013; Owaka *et al.*, 2017) included in the systematic review that were reviewed reported a varied range of prevalence of IPVp within the country, evidence that we could not project to this setting. These studies also reported mixed findings regarding the determinants of IPVp. To fill the prevalence and local determinants gaps, we sought to address the following objectives: to determine the prevalence, proportions of different types and determinants of IPVp, and to describe the adverse perinatal outcomes of survivors of IPVp, at Moi Teaching and Referral Hospital (MTRH).

A clear understanding of pregnancy-specific risk factors will aid in developing and implementing effective screening and intervention processes (James *et al.*, 2013). This study will therefore aid in understanding the problem and the predisposing and protective factors of IPVp which are crucial in pursuing diagnosis and developing preventive strategies (Louise, 2015). Efforts to formulate policies to address IPVp will also be initiated, since there is no policy direction in Kenya that addresses interruption of risk factors while being responsive to the needs of survivors. A paucity of local data on the determinants and perinatal outcomes of IPVp in this region, together with a lack of policy direction, has warranted this study.

The closest policy document available that addresses IPVp is the County Government Policy on Sexual and Gender-based Violence (SGBV) of 2017 (National Gender and Equality Commision, 2017), which was formulated by the national government to provide direction on matters of SGBV at the county level. However, this document provides general statements on prevention and response strategies for SGBV but does not include guidelines on the prevention and screening for IPVp. It would be prudent to involve a public health approach for the primary prevention of IPVp which entails documentation of the scope and magnitude of IPVp, identification of risk and protective factors and design prevention strategies grounded in social science theory for modification of the risk factors (Harvey *et al.*, 2007).

The healthcare system is one of the institutions which is likely to interact with most women during the antenatal period. This presents as a good window of opportunity for healthcare providers to identify survivors of IPVp. In addition to this, the healthcare system is still superior in diagnosing IPVp as women have been noted to admit abuse if they are questioned gently and privately by supportive healthcare workers, thus providing more accurate estimates of violence (Bacchus *et al.*, 2002).

To address the low diagnosis rates of IPVp, health care workers need to be equipped with knowledge of its local determinants. Such knowledge will act as a motivator in probing the occurrence and extent of IPVp during antenatal care.

## Methods

### Study design

This was a cross-sectional study conducted at the MTRH, one of the two national referral hospitals in Kenya.

### Study setting

Kenya is divided into regions called counties. MTRH is located in the Western Kenyan region of the country, representing at least 22 of the 47 counties of Kenya. It also serves parts of Eastern Uganda and Southern Sudan. It has a catchment population of about 24 million. The study site was opted for by virtue of it being a referral hospital which would capture a wider population that is more representative of the targeted Western region. Screening for IPVp at MTRH was not routinely taking place and therefore its prevalence and associated factors had not yet been established. Approximately 12 000 deliveries are conducted each year at the hospital.

The study population consisted of women who had delivered (vaginal deliveries and caesarean sections) between April and June 2017 and who were in the MTRH postnatal ward and mothers’ hostel. All women delivering at MTRH, including emancipated minors (those <18 years), were included in the study sample. Those who were very ill and unable to respond to the questionnaire were excluded.

### Data collection

Using the Cochran (1963) formula, based on the local prevalence of 37% of IPV among pregnant women (Makayoto *et al.*, 2013), a margin of error of 5% and 95% confidence, a sample size of 359 was arrived at as shown below.

$$\begin{array}{l} n={\left(\frac{Z_{1-\alpha /2}}{d}\right)}^{2}\times P\left(1-P\right)\\ ={\left(\frac{1.96}{0.05}\right)}^{2}\times 0.37\left(1-0.37\right)\\ =359 \end{array},$$

where *P* is the prevalence of IPV, *d* = 0.05 is the margin of error and *Z* is the quantile of the standard normal distribution corresponding to 100 × (1−*α*) %.

Systematic sampling was used to recruit the participants from the delivery register. An anticipated average population size of 1000 mothers delivering in the facility per month and an intention to carry out data collection within a period of three months were used in calculating the sampling interval. Therefore, to sample from an average population size of 3000, the sampling interval was 3000/359, which was ∼8, the denominator being the study sample size.

IPV was categorized into physical, sexual and psychological aggression using standardized definitions (Saltzman *et al.*, 2002). Data were collected through interviews and a review of clinical records by clinical research assistants (two nurses, two psychological counsellors) who were trained on sampling, data collection and confidentiality. The research assistants sampled potential respondents from the delivery register, checked for eligibility and initiated the informed consent or assent process in the wards. A separate assent form was administered to parents or guardians.

Details of all vaginal deliveries and caesarean sections were recorded in the Maternity Services Health Facility Register kept in the delivery ward of MTRH, from where the respondents were sampled. The first respondent was selected randomly from the first eight entries on the register on the first day of data collection. Subsequently, every eighth client was sampled. When a sampled client met the exclusion criteria or did not consent to take part in the study, the next client on the register was sampled. A total of 381 respondents were sampled within a period of three months. Twelve respondents that had been sampled did not consent. A sample size of 369 was finally achieved.

VAW is a phenomenon that is difficult to measure due to a variation in the types of acts considered violent by different populations and to the varied tools and methodologies used by different studies. In an attempt to minimize the methodological problems emanating from the different studies and to allow comparisons of the same studies across different cultures, the World Health Organization developed the WHO VAW instrument.

The study questionnaire consisted of four parts: (1) a researcher-designed socio-demographic and health history section; (2) a modified WHO VAW screening tool (Garcia-Moreno *et al.*, 2006); (3) intimate partner characteristics, such as age, use of alcohol and/or drugs, level of education and (4) perinatal characteristics. The WHO screening tool covered the occurrence of physical, psychological and sexual violence and was modified to include the following questions in the psychological section, as advised by a list of acts of psychological aggression compiled by Breiding et al. (2015): whether the partner isolated or confined the woman, and whether the partner prevented the woman from visiting her friends or relatives.

Any positive response to the questions on the screening tool confirmed the occurrence of IPVp.

Psychometric assessments of the WHO VAW instrument have shown that it demonstrates good internal consistency, indicating that it provides a reliable and valid measure of types of violence (Nybergh *et al.*, 2013; Marizella *et al.*, 2014). It has also demonstrated significant cross-cultural validity and reliability when comparing the IPV prevalence rates in Sweden (Nybergh *et al.*, 2013).

Data on the outcome of the pregnancy, including birth weights, 5-min Apgar score, foetal death, immediate neonatal death and gestation at birth, were obtained from patient records.

In this study, foetal death is defined as death of a foetus between 28 weeks of pregnancy and delivery, whereas immediate neonatal death is the death of a baby within 24 h of delivery. LBW was defined as weight at birth of <2500 g (WHO, 2012). A premature baby is one born before 37 weeks of pregnancy, while a preterm birth is defined as a delivery that occurred before 37 weeks of pregnancy (Blencowe *et al.*, 2012).

The Apgar score is a scoring system that rapidly provides a standardized assessment of infants after delivery. It is divided into five components: heart rate, respiratory effort, muscle tone, reflex irritability and colour. Each component is given a score of 0, 1 or 2 at 1 and 5-min intervals. A 5-min Apgar score of 7–10 is reassuring, a score of 4–6 is moderately abnormal, whereas a score of 0–3 is low (American Academy of Pediatrics, 2006).

The questionnaire underwent forward and back translation from English to Swahili according to WHO protocol (WHO, 2016a). The questionnaire was refined based on a pilot conducted in January 2017 at Uasin Gishu District Hospital with 40 respondents. Research assistants either oversaw participants filling in the questionnaires or read the questions aloud in English or Swahili for those who could not answer by themselves.

Each administered questionnaire was numbered. The gathered data were cleaned and entered into an excel spreadsheet, and encrypted to ensure confidentiality. The password was available to the principal investigator alone. Back-up of the data was done to cushion against loss. Once the data had completely been converted into the electronic database, the questionnaires were kept in a locked cabinet, and access was allowed to the principal investigator alone. They will be shredded after five years.

### Data analysis

Categorical variables were summarized using frequencies and percentages. Continuous variables were summarized using median and the corresponding interquartile range (IQR) due to a violation of Gaussian assumptions, which were assessed using the Shapiro–Wilk test and histograms. The infant birth weight was summarized using mean and standard deviation (SD).

For global comparison purposes, we conventionally reported the prevalence of physical or sexual IPVp as one indicator and psychological IPVp as the other indicator. Analyses of associations with and without adjustment for previous experience of IPV as a variable were conducted. This is because previous experience of IPV has been shown to be on the causal pathway for other determinants to IPVp, such as childhood violence or marital status of partner (Bell and Naugle, 2008).

Logistic regression modelling was used to determine factors associated with sexual or physical IPVp while adjusting for previous history of IPV. Odds ratios (OR) and 95% confidence intervals (95% CI) were used in bivariate analysis of determinants of IPVp, with significant variables further analysed in the final regression models (Tables 1 and 2) using forced entry regression technique to achieve the documented results.

**Table 1:** Determinants of physical/sexual IPVp adjusted for previous experience of IPV

Variable	Category	uOR	95% CI	aOR	95% CI
Number of sexual	1	Ref			
partners	>1	**4.31**	**1.28–14.49**	4.16	0.87–19.93
Education level	None/primary	Ref			
	Secondary	0.91	0.53–1.55	**2.34**	**1.06–5.18**
	Tertiary	**0.28**	**0.12–0.61**	0.67	0.23–2.01
Alcohol/drug abuse	No	Ref			
	Yes	**4.7**	**1.03–21.43**	2.19	0.39–12.16
Survivor of childhood violence	No	*Ref*			
	Yes	**2.09**	**1.00–4.32**	2.66	0.95–7.44
Perpetrator religion	Christian	Ref			
	Muslim	**4.00**	**1.44–11.08**	3.42	0.86–13.66
	Other	1.93	0.96–3.87	2.20	0.88–5.45
Perpetrator occupation	Self-employed	Ref			
	Formal employment	**0.49**	**0.28–0.86**	0.53	0.25–1.13
	Unemployed	1.00	0.48–2.08	1.72	0.68–4.32
	Informal employment	**0.27**	**0.08–0.95**	0.59	0.14–2.54
Perpetrator education	None/primary	Ref			
Level	Secondary	0.67	0.37–1.24	0.45	0.19–1.02
	Tertiary	**0.43**	**0.22–0.85**	0.61	0.23–1.62
Previous experience	No	Ref			
of IPV	Yes	**15.3**	**8.52–27.46**	**17.18**	**8.70–33.93**

^a^OR, adjusted odds ratio; Bold, significant variables; Ref, reference variables; uOR, unadjusted odds ratio.

**Table 2:** Determinants of psychological IPVp adjusted for previous experience of IPV

Variable	Category	uOR	95% CI	aOR	95% CI
Education level	None/primary	Ref		Ref	
	Secondary	0.83	0.49–1.38	1.45	0.74–2.84
	Tertiary	**0.38**	**0.19–0.76**	1.02	0.42–2.47
Perpetrator religion	Christian	Ref		Ref	
	Muslim	2.35	0.84–6.53	1.69	0.51–5.53
	Other	**1.97**	**1.01–3.84**	1.97	0.93–4.19
Perpetrator occupation	Self-employed	Ref		Ref	
	Formal employment	**0.42**	**0.24–0.71**	**0.50**	**0.27–0.95**
	Unemployed	0.63	0.30–1.31	0.63	0.26–1.52
	Informal employment	**0.34**	**0.12–0.95**	0.41	0.13–1.23
Previous experience of IPV	No	Ref		Ref	
	Yes	**4.10**	**2.45–6.87**	**3.76**	**2.08–6.77**
Parity	Covariate	0.19	1.04–1.37	1.07	0.89–1.28
Perpetrator education level	None/primary	Ref		Ref	
	Secondary	0.80	0.44–1.43	0.88	0.43–1.79
	Tertiary	**0.39**	**0.20–0.77**	0.72	0.29–1.81
Perpetrator income	Covariate	0.99	0.99–0.99	0.99	0.99–1.00

^a^OR, adjusted odds ratio; Bold, significant variables; CI, confidence intervals; Ref, reference variables; uOR, unadjusted odds ratio.

Characteristics of perinatal outcomes were presented in tables. A descriptive analysis of the perinatal outcomes was carried out. Analysis of associations of IPVp and perinatal outcomes could not be done, as the sample size was underpowered to provide strong analytical conclusions of these outcomes.

### Research ethics

The Institutional Research and Ethics Committee of Moi University and MTRH approved the study. All the participants gave written informed consent and their privacy and data confidentiality were maintained. Data collection was in accordance with the recommendations of WHO Ethical and Safety recommendations for intervention research on VAW (WHO, 2016b). Women who were found to have survived IPVp were offered a counselling session with a psychological counsellor.

## Results

### Participant demographics

The study approached 381 postnatal mothers and achieved a response rate of 96.9% (369 out of 381). The median age of the study participants was 25.0 years (IQR: 21.0, 31.0) compared with the median age of the country’s female population of 20.3 years. Eleven participants (3.0%) had more than one sexual partner during the pregnancy, and 23 (8.3%) were in a polygamous marriage. Eleven per cent of women in the country’s population are divorced, separated or widowed compared with 24% of the study’s population.

Of the 369 participants, 96.7% attended antenatal care, which is similar to the country’s figure of 96% of pregnant women attending antenatal care (KNBS, 2015), and 290 (81.3%) made three or more antenatal care visits.

### Partner characteristics

Of current or former partners of participants, 258 (69.9%) participants reported spouses as their intimate partners. Up to 310 (84%) of the partners were Christians, in line with the 2019 population and housing census which reported that a majority of the country’s population are Christians. A third (127 of 369) of the partners had completed college or university level of education.

### Overall perinatal characteristics

The average infant birth weight was 2.8 kg (SD: 0.7). Preterm labour and deliveries were observed in 71 (19.2%) of the 369 participants. This is higher than the country’s preterm birth rate of 12 per 100 live births as at 2010 (Project Concern, USAID, 2015). The number of foetuses who died were five (1.3%), which is comparable with the country’s stillbirth rate of 21.8/1000 live births in 2010 (WHO, 2015) (see Table 3).

**Table 3:** Perinatal outcomes of IPVp among participants

Variable	Category	Frequency	Percentage
Birth weight	Normal weight	285	77.24
	Under weight	84	22.76
Newborn maturity	Term delivery	298	80.76
	Preterm delivery	71	19.24
Foetal death	No	364	98.64
	Yes	5	1.36
Neonatal death	No	367	99.46
	Yes	2	00.54
Apgar score at 5 min	≥7	306	82.93
	<7	63	17.07

Perinatal outcomes of IPVp among women admitted to the postnatal ward of MTRH, April–June 2017.

### Perinatal characteristics of IPVp survivors

A majority (65.1%) of the survivors of IPVp had deliveries with birth weights between 2500 and 3500 g. Three of the survivors had foetal deaths but none had neonatal deaths within the initial 24 h of life. Twenty-three out of 126 (18.3%) of the survivors of IPVp had preterm deliveries, which is also higher than the country’s preterm birth rate of 12 per 100 live births as at 2010 (Project Concern, USAID, 2015). Twenty-six (20.6%) out of the 126 survivors of IPVp had Apgar scores of <7 (see Table 4).

**Table 4:** Perinatal outcomes of survivors of IPVp among women admitted to the postnatal ward of MTRH, April–June 2017

Variable	*N*	Survivor of IPVp			
		No (N = 243)		Yes (N = 126)	
		*n* or mean	% or SD	*n* or mean	% or SD
Birth weight (g)	^a^368	2.8	0.8	2.8	0.6
<1000	368	6	2.5	0	0.0
1000–1500		15	6.2	2	1.6
1500–2500		40	16.5	20	15.9
2500–3500		139	57.2	82	65.1
≥3500		43	17.7	21	16.7
Preterm labour or delivery	369	48	19.8	23	18.3
Foetal death	369	2	0.8	3	2.4
Neonatal death	369	2	0.8	0	0.0
Foetal or neonatal death	369	4	1.6	3	2.4
Apgar score					
<7	369	37	15.2	26	20.6
≥7		206	84.8	100	79.4

a Missing data (one of the birth weights was not recorded).

SD, standard deviation.

### Prevalence and types of IPVp

The overall prevalence of IPVp was 34.1% (126 of 369 participants) (Table 5). The prevalence of physical or sexual violence in pregnancy was 22.8% (84 of 369 participants) (Table 5).

**Table 5:** Prevalence of IPV during pregnancy among women admitted to the postnatal ward of MTRH, April–June 2017

Type of violence	*N*	Number of women who experienced the violence	%	95% CI
Physical	369	54	14.6	11.4–18.6
Sexual	369	48	13.0	9.9–16.8
Psychological	369	101	27.4	23.0–32.2
Physical or sexual	369	84	22.8	18.7–27.3
Physical, sexual or psychological	369	126	34.1	29.5–39.2

CI, confidence intervals.

Up to 46 (36.5%) of the survivors of IPVp were slapped or had something that could hurt thrown at them. In addition, 38 survivors (30.2%) had sex when not in the mood due to fear. Upon further analysis, psychological violence emerged as the topmost type of IPVp amongst the survivors, at 27.4% (101 of 369). The least frequent type of IPVp to occur was sexual violence at 13% (48 of 369). Estimates of previous experience of IPV were higher than IPV during pregnancy in all types of IPVp (see Table 6).

**Table 6:** Previous experience of IPV before pregnancy

Type of violence	*N*	Number of women who experienced the violence	%	95% CI
Physical	369	59	16.9	12.6–20.1
Sexual	369	46	12.5	9.5–16.3
Psychological	369	114	30.9	26.4–35.8
Physical or sexual	369	84	22.8	18.8–27.3
Physical, sexual or psychological	369	133	36.0	31.3–41.1

Previous (before the pregnancy) experience of various IPV among women admitted to the postnatal ward of MTRH, April–June 2017.

**Figure 2:**
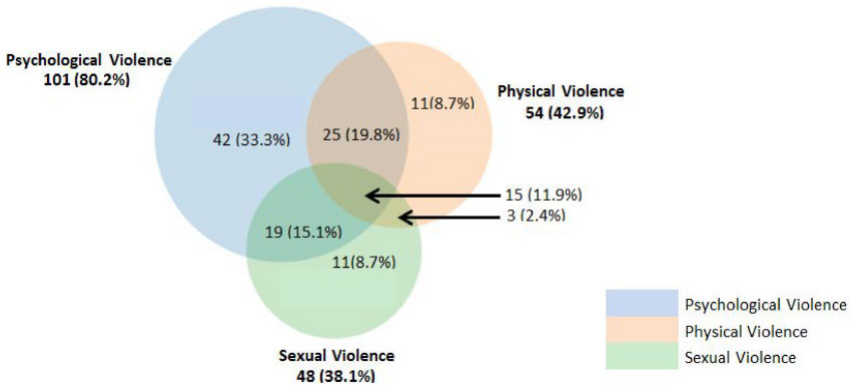
Intersections of the types of IPVp among survivors of IPVp in women giving birth at MTRH, April to June, 2017

Refer to Table 7 for prevalence figures for ever having experienced IPV, whether during pregnancy or not, and Supplementary Table S9 for further results on the different types of IPVp. The intersections of the different types of IPVp among the survivors of IPVp show that a majority (19.8%) of these women had an overlap of physical and psychological violence, as shown in Figure 2.

**Table 7:** Prevalence figures for ever having experienced IPV, whether during pregnancy or not

Type of violence	N	Number of women who experienced the violence	%	95% CI
Physical	369	83	22.5	18.5–27.5
Sexual	369	62	16.8	13.3–21.0
Psychological	369	142	38.5	33.6–43.6
Physical or sexual	369	114	30.9	26.4–35.8
Physical, sexual or psychological	369	164	44.4	39.4–49.6

Prevalence of ever having experienced IPV before or during the pregnancy by women admitted to the postnatal ward of MTRH, April–June 2017.

### Determinants of physical or sexual IPVp

Evidence on bivariate analysis of determinants of physical or sexual IPVp using logistic regression (Table 8) demonstrated that participants who experienced physical or sexual IPVp were more likely to have more than one sexual partner (unadjusted odds ratio [uOR]: 4.31), to be survivors of childhood violence (uOR 2.09) and to have previous experience of IPV (uOR 15.3).

**Table 8:** Intersections of the types of IPVp among women giving birth at MTRH, April to June, 2017

Variable	Category	uOR	95% CI
Age	Covariate	0.998	0.96–1.03
Marital status	Single	Ref	
	Married	1.61	0.87–2.99
Number of sexual	1	Ref	
partners	>1	4.31*	**1.28–14.49**
Education level	None/primary	Ref	
	Secondary	0.91	0.53–1.55
	Tertiary	0.28*	**0.12–0.61**
Income	Covariate	0.999	0.99–1.00
Living with	Partner	Ref	
	Parents	0.91	0.50–1.65
	Other	1.05	0.40–2.75
Alcohol/drug abuse	No	Ref	
	Yes	4.7*	**1.03–21.43**
Survivor of childhood violence	No	Ref	
	Yes	2.09*	**1.00–4.32**
Mother survivor of	No	Ref	
	Yes	1.90	0.92–3.90
Perpetrator age	Covariate	1.03	0.99–1.06
Perpetrator relationship	Other	Ref	
	Spouse	0.85	0.51–1.42
Perpetrator religion	Christian	Ref	
	Muslim	4.00*	**1.44–11.08**
	Other	1.93	0.96–3.87
Perpetrator occupation	Self-employed	Ref	
	Formal employment	0.49*	**0.28–0.86**
	Unemployed	1.00	0.48–2.08
	Informal employment	0.27*	**0.08–0.95**
Current pregnancy plan	Planned	Ref	
	Not planned	1.08	0.66–1.78
Previous experience of IPV	No	Ref	
	Yes	15.3*	**8.52–27.46**
Parity	Covariate	1.10	0.95–1.27
Perpetrator education	None/primary	Ref	
Level	Secondary	0.67	0.37–1.24
	Tertiary	0.43*	**0.22–0.85**
Perpetrator income	Covariate	1.00	0.99–1.00

Determinants of physical/sexual IPVp among women admitted to the postnatal ward of MTRH, April–June 2017.

Bolded uOR* (unadjusted odds ratio) are the significant variables.

CI, confidence intervals; Ref, reference variable.

Participants who had higher levels of education (tertiary level) were less likely to suffer from physical or sexual IPVp (uOR 0.28) than those with secondary and primary levels of education. This association remained significant across the crude, unadjusted and adjusted analyses (see Tables 1, 8 and 9).

**Table 9:** Determinants of physical/sexual IPVp not adjusted for previous experience of IPV

Variable	Category	uOR	95% CI	aOR	95% CI
Number of sexual	1	*Ref*			
partners	>1	**4.31**	**1.28–14.49**	**5.25**	**1.47–18.71**
Education level	None/primary	Ref			
	Secondary	0.91	0.53–1.55	1.15	0.61–2.18
	Tertiary	**0.28**	**0.12–0.61**	**0.33**	**0.13–0.87**
Alcohol/drug abuse	No	*Ref*			
	Yes	**4.7**	**1.03–21.43**	4.25	0.89–20.33
Survivor of childhood violence	No	*Ref*			
	Yes	**2.09**	**1.00–4.32**	2.10	0.91–4.85
Perpetrator religion	Christian	*Ref*			
	Muslim	**4.00**	**1.44–11.08**	**4.45**	**1.42–13.91**
	Other	1.93	0.96–3.87	1.69	0.78–3.66
Perpetrator occupation	Self-employed	*Ref*			
	Formal employment	**0.49**	**0.28–0.86**	0.58	0.31–1.10
	Unemployed	1.00	0.48–2.08	1.20	0.55–2.65
	Informal employment	**0.27**	**0.08–0.95**	0.30	0.08–1.11
Perpetrator education	None/primary	*Ref*			
Level	Secondary	0.67	0.37–1.24	0.59	0.29–1.19
	Tertiary	**0.43**	**0.22–0.85**	0.75	0.32–1.74

^a^OR, adjusted odds ratio; Bold, significant variables; Ref, reference variables; uOR, unadjusted odds ratio.

When adjusting for the risk factors but not for previous experience of IPV, having multiple sexual partners (adjusted odds ratio [aOR]: 5.25; 95% CI: 1.47, 18.71) and having a partner who is a Muslim (aOR 4.45) were consistently associated with a higher likelihood of IPVp in both the unadjusted and adjusted models (see Table 9).

When adjusting for previous experience of IPV in addition to the other risk factors in the regression model (Table 1), evidently having a secondary level of education for the woman was associated with more than twice the odds (aOR: 2.34, 95% CI: 1.06, 5.18) of suffering from physical or sexual IPV than those with a lower level of education (none or primary) and those with a higher level of education (tertiary) (aOR: 0.67, 95% CI: 0.23, 2.01). Having a previous experience of IPV was also associated with >17 times increased odds of suffering from physical/sexual IPVp than not having a previous history of IPV (aOR: 17.18, 95% CI: 8.70, 33.93).

### Determinants of psychological IPVp

Evidence on bivariate analysis of determinants of psychological violence (Tables 10 and 1) showed that a woman with tertiary level of education is protected from psychological violence (uOR 0.38, 95% CI 0.19–0.76) compared with women with lower levels of education. Perpetrators with a tertiary level of education were also found to be less likely to inflict psychological IPVp on women (uOR 0.39; 95% CI 0.20–0.77). A previous experience of IPV for the woman and a religion other than Christianity or Islam for the perpetrator were also likely to be associated with the occurrence of psychological IPVp.

**Table 10:** Bivariate analysis of determinants of psychological IPVp

Variable	Category	uOR	95% CI
Age	Covariate	1.01	0.97–1.04
Marital status	Single	Ref	
	Married	1.29	0.74–2.25
Number of sexual partners	1	Ref	
	>1	2.27	0.67–7.62
Education level	None/primary	Ref	
	Secondary	0.83	0.49–1.38
	Tertiary	0.38*	**0.19–0.76**
Income	Covariate	0.99	0.99–1.00
Living with	Partner	Ref	
	Parents	1.00	0.57–1.74
	Other	1.03	0.41–2.58
Alcohol/drug abuse	No	Ref	
	Yes	2.02	0.44–9.18
Survivor of childhood violence	No	Ref	
	Yes	1.79	0.88–3.67
Mother survivor of violence	No	Ref	
	Yes	1.62	0.80–3.29
Perpetrator age	Covariate	1.02	0.99–1.05
Perpetrator relationship	Other	Ref	
	Spouse	0.83	0.51–1.35
Perpetrator religion	Christian	Ref	
	Muslim	2.35	0.84–6.53
	Other	1.97*	**1.01–3.84**
Perpetrator occupation	Self-employed	Ref	
	Formal employment	0.42*	**0.24–0.71**
	Unemployed	0.63	0.30–1.31
	Informal employment	0.34*	**0.12–0.95**
Current pregnancy plan	Planned	Ref	
	Not planned	1.53	0.96–2.43
Previous experience of IPV	No	Ref	
	Yes	4.10*	**2.45–6.87**
Parity	Covariate	0.19	**1.04–1.37**
Perpetrator education	None/primary	Ref	
Level	Secondary	0.80	0.44–1.43
	Tertiary	0.39*	**0.20–0.77**
Perpetrator income	Covariate	0.99	0.99–0.99

Determinants of psychological IPVp among women admitted to the postnatal ward of MTRH, April–June 2017.

Bolded uOR* (unadjusted odds ratio) are the significant variables.

CI, confidence intervals; Ref, reference variable.

**Table 11:** Determinants of psychological IPV not adjusted for previous experience of IPV

Variable	Category	uOR	95% CI	aOR	95% CI
Education level	None/primary	Ref		Ref	
	Secondary	0.83	0.49–1.38	1.22	0.64–2. 23
	Tertiary	**0.38**	**0.19–0.76**	0.76	0.32–1.79
Perpetrator religion	Christian	Ref		Ref	
	Muslim	2.35	0.84–6.53	2.22	0.73–6.76
	Other	**1.97**	**1.01–3.84**	1.91	0.92–3.92
Perpetrator occupation	Self-employed	Ref		Ref	
	Formal employment	**0.42**	**0.24–0.71**	**0.52**	**0.28–0.95**
	Unemployed	0.63	0.30–1.31	0.64	0.27–1.52
	Informal employment	**0.34**	**0.12–0.95**	**0.28**	**0.09–0.84**
Parity	Covariate	**0.19**	**1.04–1.37**	1.15	0.97–1.36
Perpetrator education	None/primary	Ref		Ref	
Level	Secondary	0.80	0.44–1.43	0.89	0.45–1.77
	Tertiary	**0.39**	**0.20–0.77**	0.68	0.28–1.65
Perpetrator income	Covariate	0.99	0.99–0.99	0.99	0.99–1.00

aOR, adjusted odds ratio; Bold, significant variables; CI, confidence intervals; Ref, reference variables; uOR, unadjusted odds ratio.

After adjusting for any previous experience of IPV, the perpetrator having formal employment was protective of psychological IPVp (aOR 0.50, 95% CI 0.27–0.95), whereas having a previous experience of IPV was strongly associated with current occurrence of psychological IPVp (aOR 3.76, CI 2.08–6.77) (see Table 2).

## Discussion

### Prevalence of IPV

The prevalence of overall IPVp (34.1%) and physical/sexual IPVp (22.8%) found in this study provides point estimates of the occurrence of IPVp in Western Kenya. It falls within the global lifetime prevalence of IPV (15–71%) and is comparable with the 37% IPVp prevalence found in Kisumu District Hospital (Makayoto *et al.*, 2013) which is located in the same region as MTRH. However, the prevalence finding differs from other estimates, such as the prevalence of IPVp in antenatal attendees (66.9%) found in West Pokot in 2017 (Owaka *et al.*, 2017). This difference may be explained by differences in social, cultural and economic environments. Whereas MTRH is located in an urban setting, West Pokot is a rural setting with a different ethnic majority, and thus has differences in social norms surrounding violence. Many participants in the West Pokot study conducted by Owaka et al. (2017) described IPV as a consistent and unchangeable aspect of local culture. In addition, differences may be explained by divergent study designs.

Owaka *et al.* (2017) employed a qualitative and quantitative cross-sectional study design and utilized a stratified, two-stage random sampling of women from 11 different facilities of West Pokot, thus representing a wider community which is different from the cross-sectional systematic sampling of women who had delivered from a single facility that this study employed.

Despite all the women in the study having a high utilization of antenatal care services (96.7%), it is also known that women who are survivors of IPVp have a 25% decreased odds of utilizing healthcare services (Musa *et al.*, 2019). This could also impact on the prevalence, thus yielding lower estimates in hospital settings than in community settings.

The prevalence of IPVp found in this study is higher than that of some risk factors of poor maternal pregnancy outcomes, including pre-eclampsia (0.3%) (Megumi *et al.*, 2017) and gestational diabetes mellitus (2.9%) (Pastakia *et al.*, 2017). This emphasizes the importance of addressing IPVp as a public health concern.

### Types of IPVp

Psychological violence emerged as the most prevalent type of IPVp in the current pregnancy (27.4%) and even during previous experiences of IPV (30.9%). This finding is comparable with results from Kenya and South Africa which also found that psychological violence was the most common (Hoque *et al.*, 2009; Makayoto *et al.*, 2013; Groves *et al.*, 2015; Owaka *et al.*, 2017). Psychological violence is mostly subtle and hidden. This possibly encourages perpetrators to inflict this type of violence, as the lack of evidence will protect them from being exposed. The high levels of psychological IPVp are of concern, since this type of violence has been shown to be associated with postnatal depression independent of physical or sexual violence (Ludermir *et al.*, 2010).

### Determinants of IPVp

Our results confirm past experience of violence as a strong risk factor for IPVp. This is shown by the analyses of either physical or sexual (aOR: 17.18; 95% CI: 8.70–33.93) and psychological (aOR 3.76; 95% CI 2.08–6.77) IPVp from this study and other reviewed articles (Shamu *et al.*, 2011; WHO, 2012). Abuse before pregnancy is the strongest risk factor for predicting IPVp. Pregnant women whose partners abused them before the pregnancy were found to have greater odds of being abused during pregnancy than women with no history of abuse. A previous experience of IPV is a background factor of the social learning theory of IPV and contributes to the development and maintenance of aggression leading to the occurrence of IPVp (Bell and Naugle, 2008).

Participants who had a college or university education had a reduced risk of physical or sexual IPVp (aOR 0.67, 95% CI, 0.23–2.01). This is in keeping with what was reported by Shamu *et al.* (2011), where three studies found a strong association between a woman’s low level of education and occurrence of IPVp. Mezey and Bewley (1997), Karamagi *et al.* (2006) and Hoque *et al.* (2009) also found significant associations between the two.

An advanced level of education therefore is associated with a reduced odds of being a victim of physical or sexual IPVp. This could be explained by Marium's (2014) suggestion that a woman’s schooling has a positive impact on her spousal relationship because the communication gap between the husband and the wife is reduced (Marium, 2014). This results in protection of the woman from physical/sexual IPVp.

From the bivariate analyses, partner employment (formal or informal employment) was found to be protective of both physical or sexual and psychological IPVp (see Tables 8 and 10). In addition, in the adjusted analysis of psychological IPVp, partners with formal employment had a 50% reduced odds (aOR 0.50; 95% CI 0.27–0.95) of inflicting psychological IPVp (see Table 2). Despite competing theories (Leung *et al.*, 1999; Martin *et al.*, 2004; Swanberg *et al*, 2005) about the effect of employment on violence, partner employment was associated with reduced IPVp in this study.

The ecological framework of IPV employed in this study is multi-contextual. The factors exhibited at each level, i.e. individual, relationship, community and societal, are backed by the known theories of IPV which include the situational model, the background model, power theory, social learning theory and feminist theory (Burelomova *et al.*, 2018). Thus factors emanating from this study will directly lead to the development of preventive measures which indirectly address target these theories.

### Perinatal outcomes of IPVp

The study’s preterm birth rates for survivors of IPVp were found to be higher than the country’s preterm birth rates. Preterm births and their complications are one of the major causes of direct neonatal mortalities, and are responsible for 35% of the world’s neonatal deaths each year (Blencowe *et al.*, 2012). In an effort to prevent preterm births and eventually achieve Sustainable Development Goal number 3.2, which aims at reducing neonatal mortality by 2030, it may be prudent to address the factors that propagate the occurrence of IPVp through primary and secondary preventive interventions.

## Study limitations

Recall bias—The study relied on the participants to recall certain events which had happened in the past. This created a level of bias as there is a likelihood that not all events could be remembered.

Social desirability bias—There is a possibility that the respondents answered some questions in a manner that was viewed as favourable to the study (over-reporting good behaviour or underreporting undesirable behaviour).

Confounding bias—Other factors not looked for in the study could have positively or negatively influenced occurrence of the determinants; e.g. societal factors which also influenced the occurrence of IPVp, and other factors e.g. congenital anomalies which influenced occurrence of the perinatal outcomes of IPVp.

The study gives a facility prevalence of IPVp. Given that there is a 25% reduced odds of survivors of IPVp attending healthcare services, this estimate may not be a true reflection of the prevalence in the community.

### Implications for policy and practice

The implications of the study results are that the prevalence rates of IPVp for women in Western Kenya remain high. Therefore, urgent policy action is required. Universal screening for IPVp during pregnancy has been suggested for Columbia in South Carolina (Coker *et al.*, 2004). This approach should be considered for women seeking antenatal healthcare services in Kenya. Its implementation would require training of healthcare workers and provision of clinical guidelines for screening and diagnosis, as well as reporting of IPVp. Fundamentally, this approach would support the development of a surveillance system for IPVp, which is a component of the Centers of Disease Control's (CDC) five-year vision for IPV (Ramsay *et al.*, 2009; CDC Strategic Direction for Intimate Partner Violence Prevention).

In countries where implementation of universal screening is not feasible, policies to advocate for identification of survivors of physical/sexual IPVp by further probing of women presenting with the risk factors emanating from this study (previous experience of IPV, and a secondary level of education) may be used by healthcare providers to aid the diagnosis of women suffering from IPVp. Hamberger *et al.* (2015), in their efforts to address barriers to routine screening (e.g. time constraints) using a systems level intervention, advocate for screening through asking about health-related risk factors of IPV.

Programme specialists need to consider interventions, programmes and policies that promote respectful, nonviolent relationships as well as interrupt the development of IPV perpetration to prevent IPVp (CDC Strategic Direction for Intimate Partner Violence Prevention). Decision makers should consider interventions that address risk factors identified in this study. Such interventions would aid in the identification and prevention of IPV prior to pregnancy, since interventions which are made after pregnancy may be late as the adverse maternal and perinatal outcomes will have already been triggered.

Interventions to prevent psychological IPVp and respond to IPVp survivors’ needs should be formulated so as to prevent its associated effects such as postnatal depression (Ludermir *et al.*, 2010).

Clinical service delivery points need to incorporate counselling with provision of safety planning advice (Ramsay *et al.*, 2009) and referral of survivors of IPVp to shelters, law enforcers and legal advisors. These approaches should be incorporated into the country’s clinical guidelines and training manuals to be dispatched to stakeholders. Legal reforms on the repercussions for perpetrators and access to justice for survivors of IPVp should be well stipulated.

Policies and programmes can also include innovative approaches like home visitation programmes. Van-Parys *et al.* (2014), in a systematic review of interventions of IPV and pregnancy, advocate for home visitation programmes for families at risk of IPVp which have shown statistically significant decreases in IPVp in their study.

Public awareness campaigns, as well as education and training of the community, are key in the prevention of IPVp as they inform and influence individuals' attitudes on the problem and aid in improving the communication and relationship skills of the couple (Harvey *et al.*, 2007).

## Conclusion

Despite continued concerns about under-reporting, the estimated prevalence of IPVp in Western Kenya remains high. Psychological IPVp is a concern due to its frequency. Previous experience of IPV is a strong predictor of IPVp, and strategies to interrupt its occurrence should be advocated for. Women's empowerment through attaining higher levels of education, and partners having employment, will encourage lower levels of IPVp.

Urgent policy and practice actions are required in Kenya to address this ongoing public health concern. Screening approaches are needed to identify women at risk and/or surviving IPVp and refer them to adequate services as well as establish a surveillance system for IPVp. Policies, practices and programmes targeting preventive interventions that address the promotion of respectful, nonviolent relationships, interrupt the development of perpetration and address risk factors are required (Ramsay *et al.*, 2009).

Structural and policy approaches have promising results in primary prevention of IPVp. Such approaches include fostering gender equality and women’s empowerment, legal reform and strengthening criminal justice responses, integrating IPVp into other programmes and improving the safety of physical environments (Harvey *et al.*, 2007).

A coordinated multisectoral approach is essential in addressing IPVp (Aura, 2017).

##  

*Conflict of interest statement*. None declared.

## Supplementary Material

czaa103_Supplementary_Materials
