# Supplementary material for: The magnitude of intimate partner violence during pregnancy in Eldoret, Kenya: exigency for policy action
Source: Health Policy Plan. 2020 Nov 9;35(Suppl 1):i7–i18. doi: 10.1093/heapol/czaa103 (PMC7751018; doi:10.1093/heapol/czaa103)
Supplement: czaa103_Supplementary_Materials [file czaa103_supplementary_materials.docx]

**SUPPLEMENTARY MATERIALS**

**Table 9**

**Experiences of Intimate Partner Violence in pregnancy as per the modified WHO Violence Against Women Instrument*.**

| **Type of Intimate Partner Violence in Pregnancy** |  | **(N=126)**  **n** |  | **(%)** |  |
| --- | --- | --- | --- | --- | --- |
| **Physical** |  | 54 |  | (42.8) |  |
| Slapped or thrown something at that could hurt |  | 46 |  | (36.5) |  |
| Pushed or shoved |  | 32 |  | (25.4) |  |
| Hit with a fist or something else that could hurt |  | 19 |  | (15.1) |  |
| Kicked or dragged or beaten up |  | 16 |  | (12.7) |  |
| Chocked or burnt on purpose |  | 5 |  | (3.9) |  |
| Threatened to use or actually used a gun, knife or another weapon to assault |  | 8 |  | (6.3) |  |
| **Sexual** |  | 48 |  | (38.0) |  |
| Physically forced to have sex when not in the mood |  | 21 |  | (16.7) |  |
| Had sex when not in the mood due to fear |  | 38 |  | (30.2) |  |
| Forced to do sexually degrading or humiliating acts |  | 24 |  | (19.0) |  |
| **Psychological** |  | 101 |  | (80.2) |  |
| Insulted or made to feel bad |  | 77 |  | (61.1) |  |
| Belittled or humiliated in front of other people |  | 47 |  | (37.3) |  |
| Scared or intimidated on purpose |  | 29 |  | (23.0) |  |
| Threated to be hurt you or hurt someone you care about |  | 17 |  | (13.5) |  |
| Ever been isolated or confined |  | 44 |  | (34.9) |  |
| Prevented from visiting friends or relatives |  | 52 |  | (41.2) |  |

**Experiences of Intimate Partner Violence in pregnancy among women admitted to the postnatal ward of Moi Teaching and Referral Hospital, April to June 2017.*

*WHO = World Health Organization*

**APPENDIX**

**QUESTIONNAIRE/DATA COLLECTION FORM**

**Participants’ Serial Number_________**

**A. DETAILS OF CURRENT ADMISSION**

1. Date of Delivery __________________
2. Gestation by dates____________ LNMP_____________EDD_____________
3. Diagnosis at admission _____________________
4. Diagnosis after delivery_____________________
5. Mode of Delivery _______________________________________

**B. DEMOGRAPHICS**

1. Age (years): ___________
2. Marital status

[ ] Single [ ] Married (monogamous/ polygamous) [ ] Divorced/Separated

Number of years in marriage _______________

Number/s of current sexual partner/s in the just concluded pregnancy __________

1. Religion: [ ] Catholic [ ] protestant [ ] Muslim Others__________________________
2. Occupation: [ ] Formal employment [ ] Unemployed [ ] Self- employment

[ ] Informal employment

1. Amount of income earned per month __________________
2. Highest level of education: [ ] no formal education [ ] Pre-primary [ ] Primary

[ ] Secondary [ ] Certificate/diploma [ ] degree and above [ ] Unknown

**C. PAST MEDICAL AND SURGICAL HISTORY**

12. History of any chronic illness _____________________________________

**D. FAMILY SOCIAL HISTORY**

13. Living arrangements: With; [ ] Parents [ ] Partner [ ] Other family members

[ ] Others______________________

1. Alcohol use or drug use: [ ] Yes [ ] No

If yes which drugs: [ ] Alcohol [ ] Cigarettes [ ] Bhang [ ] Miraa / Khat [ ] Others__________

Was alcohol or drug intake initiated in this pregnancy? _________

Has the frequency/amount of use of alcohol/drugs increased with onset of IPV in pregnancy? _____________

15. Were you a victim of any form of violence as a child? [ ] yes [ ] No

If yes, which form was it? [ ] physical [ ] sexual [ ] psychological

1. Was your mother a victim of any form of violence (Physical, Sexual or psychological)? [ ] Yes [ ] No

**E. OBSTETRIC HISTORY**

17. Gravidity: ____________________

18. Parity: ______________________

19. Antenatal clinic attendance [ ] YES [ ] NO

If YES, Date of first ANC visit_____________________

Number of visits [ ] 1 [ ] 2 [ ] 3 [ ] 4 and above

1. HIV test: HIV [ ] Negative [ ] HIV Positive

If HIV positive, was HIV diagnosed during this pregnancy or before? ___________________________

1. Was the current pregnancy planned [ ]YES [ ] NO

F. **OUTCOME OF DELIVERY (PERINATAL OUTCOME)**

a) Preterm delivery [ ]

b) Fetal death [ ]

c) Early neonatal death [ ]

d) Birth weight [ ]

5 minute APGAR Score _____________

**G. MODIFIED WHO VIOLENCE AGAINST WOMEN STUDY INSTRUMENT**

**1) Physical violence (tick any)**

In the most recent pregnancy, did your husband/ex-husband/boyfriend or ex-boyfriend

1. Slap you or throw something at you that could hurt you? [ ]
2. Push or shove you? [ ]
3. Hit you with his fist or something else that could hurt you? [ ]
4. Kick you, drag you or beat you up? [ ]
5. Chock or burn you on purpose? [ ]
6. Threaten to use or actually use a gun, knife or another weapon against you? [ ]

**Had he ever done so before the pregnancy? ___________**

**2) Sexual violence (tick any)**

In the most recent pregnancy, did your husband/ex-husband/boyfriend or ex-boyfriend.

a) Physically force you to have sexual intercourse when you did not want to? [ ]

b) Did you ever have sexual intercourse when you did not want because you were afraid of what he might do? [ ]

c) Has he forced you to do something sexual that you found degrading or humiliating? [ ]

**Had he ever done so before the pregnancy? _____________**

**3) Psychological violence (tick any)**

In the most recent pregnancy, did your husband/ex-husband/boyfriend or ex-boyfriend

a) Insult you or make you feel bad about yourself? [ ]

b) Belittle or humiliate you in front of other people? [ ]

c) Do things to scare or intimidate you on purpose? [ ]

d) Threaten to hurt you or someone you care about? [ ]

e) Isolate or confine you? [ ]

f) Prevent you from visiting your friends or relatives [ ]

**Had he ever done so before the pregnancy? ____________**

**H. PERPETRATOR OR PARTNER CHARACTERISTICS**

**a) Who was the perpetrator or partner?**

Spouse [ ] boyfriend [ ] ex-partner [ ] family members [ ] others [ ]

**b)** **Age of perpetrator / partner**: ____________________

**c) Religion of perpetrator/ partner**: Christian [ ] Muslim [ ] others [ ]

**d) Employment status**: [ ] unemployed [ ] formal employment [ ] self-employment

[ ] informal employment

**e) Education level**: [ ] No formal education [ ] pre-primary level [ ] Primary level

[ ] Secondary [ ] certificate/diploma level [ ] degree or more [ ] unknown

1. **Alcohol or use of other drugs by perpetrator** [ ] YES [ ] NO

**If yes, which type? ____________**

1. **Amount of income earned per month _____________________**
